# Structure and Function of Lactate Dehydrogenase from Hagfish

**DOI:** 10.3390/md8030594

**Published:** 2010-03-15

**Authors:** Yoshikazu Nishiguchi, Nobue Ito, Mitsumasa Okada

**Affiliations:** 1 Faculty of Pharmaceutical Sciences, Toho University, Japan; 2 Faculty of Science, Toho University, Japan; E-Mails: itonobue@chem.sci.toho-u.ac.jp (N.I.); mokada65@kem.biglobe.ne.jp (M.O.)

**Keywords:** hagfish, lactate dehydrogenase, high-pressure adaptation, evolutionary medicine

## Abstract

The lactate dehydrogenases (LDHs) in hagfish have been estimated to be the prototype of those in higher vertebrates. The effects of high hydrostatic pressure from 0.1 to 100 MPa on LDH activities from three hagfishes were examined. The LDH activities of *Eptatretus burgeri*, living at 45–60 m, were completely lost at 5 MPa. In contrast, LDH-A and -B in *Eptatretus okinoseanus* maintained 70% of their activities even at 100 MPa. These results show that the deeper the habitat, the higher the tolerance to pressure. To elucidate the molecular mechanisms for adaptation to high pressure, we compared the amino acid sequences and three-dimensional structures of LDHs in these hagfish. There were differences in six amino acids (6, 10, 20, 156, 269, and 341). These amino acidresidues are likely to contribute to the stability of the *E. okinoseanus* LDH under high-pressure conditions. The amino acids responsible for the pressure tolerance of hagfish are the same in both human and hagfish LDHs, and one substitution that occurred as an adaptation during evolution is coincident with that observed in a human disease. Mutation of these amino acids can cause anomalies that may be implicated in the development of human diseases.

## 1. Introduction

The deep sea is characterized by low temperature (1–4 °C), extremely high hydrostatic pressure, lack of sunlight, and relatively low influx of utilizable organic material derived from primary production in surface waters. Among such environmental factors, hydrostatic pressure is thought to have the greatest influence on the vertical distribution of organisms and speciation in the deep seas [1–3] and on the formation of protein complexes, e.g., enzyme–substrate or protein–protein interactions [4]. Many previous studies reported proteins from deep-sea fishes that function at high pressure [4–6], and hypothetical models of protein adaptation to deep-sea pressure have been proposed [4]. However, the primary structures of those proteins have not been determined in detail [3]. The effects of pressure on the K_m_ values of many enzymes from shallow- and deep-dwelling marine invertebrates and fishes were studied [5]. Malate dehydrogenase from organisms adapted to pressure of 0.1 MPa increased with additional pressure, resulting in a concomitant increase in the K_m_ values for nicotinamide adenine dinucleotide (NADH) [7]. The α-skeletal actin cDNA from *Coryphaenoides* sp. was cloned and sequenced, and the specific amino acid substitutions responsible for the adaptation of α-actin to high pressure were elucidated [3]. The kinetic properties and amino acid sequences of lactate dehydrogenase (LDH) expressed in skeletal muscle [L-LDH: NAD oxidoreductase, EC 1.1.1.27; (LDH-A_4_)] were compared among orthologs from congeners of Pacific damselfish, and temperature-adaptive changes in LDH-A_4_ structure and function were found [8]. When the kinetic properties and amino acid sequences of LDH in heart muscle (LDH-B_4_) from two cod species were compared, pressure-adaptive changes in LDH-B_4_ structure and function were observed. The results of circular dichroism spectroscopic analysis pointed to protein unfolding as the cause of inactivation [9]. In those studies, however, the amino acids responsible for the adaptation were not identified.

We previously reported the kinetic properties and amino acid sequences of LDH-A_4_ from three hagfishes inhabiting different depths [10]. The activity of LDH-A from the shallow-sea hagfish *Eptatretus burgeri* was almost completely lost when subjected to pressure of 50 MPa for three minutes, while that from the deep-sea hagfish *Eptatretus okinoseanus* did not change when subjected to the same conditions, and thus a pressure-dependent change in LDH-A_4_ structure was proposed [11]. In this study, we investigated the pressure-adaptive mechanism of LDHs from hagfish and considered the relationship between this mechanism and human disease.

## 2. Results and Discussion

**Figure 1:**
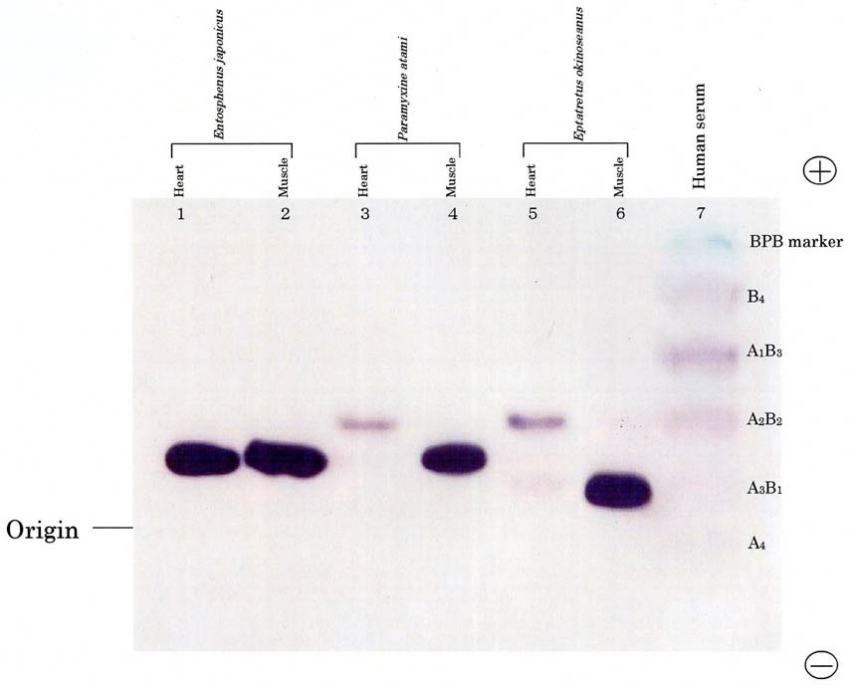
Patterns of the Cyclostomata LDH isozymes. LDH-A_4_ bands are detected in skeletal muscle from all cyclostomata examined and in the *E. japonica* heart, whereas B_4_ bands are expressed only in hearts from hagfish. Note that labels (A_4_, A_3_B_1_, A_2_B_2_, A_1_B_3_, and B_4_) indicate five human tetrameric isozymes and do not correspond to Cyclostomata LDH isozymes. Reproduced with permission from the Zoological Society of Japan [10].

The phylogenies of hagfish and lampreys in the Cyclostomata are not yet clear-cut. In this study, we compared their isozymes using electrophoresis. Figure 1 shows the electrophoretic patterns of LDH isozymes from the skeletal muscle and heart of two hagfishes, *E. okinoseanus* and *Paramyxine atami*, and a lamprey, *Entosphenus japonica*. Lampreys are also jawless fish, and their evolutionary relationship with hagfish is under debate. The skeletal muscle of the three species had the A_4_ isozyme (Figure 1, lanes 2, 4, and 6). The B_4_ isozyme was expressed in the hearts of the two hagfish (lanes 3 and 5), but not in the lamprey (lane 1). Whereas human serum LDH had heteroisozymes (lane 7), such as A_1_B_3_, A_2_B_2_, and A_3_B_1_, hagfish LDHs did not. Figure 1 indicates that hagfish diverged after lampreys in the evolution of vertebrates.

**Figure 2:**
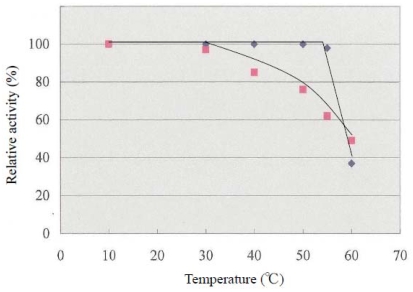
Thermal stability of LDH from hagfish. ▪: *E. okinoseanus* LDH-A_4_, ●: *E. burgeri* LDH-A_4_.

As shown in Figure 2, the thermal stability of LDH activity was examined in the reaction from pyruvic acid to lactic acid at temperatures ranging from 10 to 60 °C during 30-min incubation. At 60 °C, the activity of LDH *from E. okinoseanus and E. burgeri* decreased to about 40% of the original value.

The activity of *E. burgeri* remained constant with heat treatment in the range from 10 °C to 55 °C, whereas that of *E. okinoseanus* gradually decreased. Thus, the heat stability of LDH-A_4_ from the hagfish differs depending on the depth of their habitats. The reason why LDHs from the hagfish show these thermophilic properties is not yet clear. Johns and Somero reported that the heat stability of Pacific damselfish LDH was achieved by amino acid substitution of residue 219 from Thr to Ala [8]. In the three hagfish LDHs examined in this study, amino acid 219 was Leu and not relevant to the differences in heat stability among them. None of the additional region that is specific and common to hagfish LDHs (amino acids 220–227), is responsible for heat stability.

**Figure 3:**
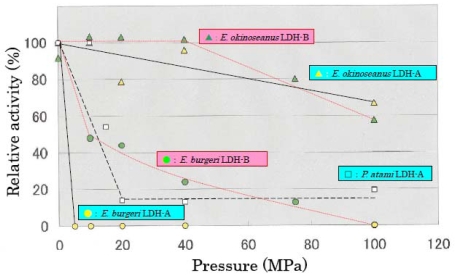
Effects of hydrostatic pressure on LDHs from hagfish *E. okinoseanus*, *E. burgeri* and *P. atami.*

The effects of high hydrostatic pressure from 0.1–100 MPa on LDH activities from the three hagfishes were examined using high-pressure photometry at the Japan Agency for Marine-Earth Science and Technology. Figure 3 shows the effects of hydrostatic pressure on LDH activities. LDH-A and -B from *E. okinoseanus*, living at a depth of 1000 m, were highly active at high pressure of 100 MPa, maintaining 70% of that observed at 0.1 MPa. In contrast, the activities of LDH-A and -B from *P. atami*, living at 250–400 m, decreased to 55% at 15 MPa, and those from *E. burgeri*, living at 45–60 m, were completely lost at 5 MPa. These results show that the deeper the habitat, the greater the tolerance to pressure.

We compared the electrophoretic patterns of the LDH-As from *E. okinoseanus* and *E. burgeri* under high-pressure conditions. The tetrameric structure of LDH-As from *E. okinoseanus* did not change at 50 MPa. In contrast, almost all LDH tetramers from *E. burgeri* dissociated to dimmers and monomers at 50 MPa but reverted to tetramers at 0.1 MPa. These results show that the dissociation of tetramers caused the inactivation of *E. burgeri* LDH. The mechanism of the slight gradual inactivation of *E. okinoseanus* LDH at high pressure differs and is probably due to the metamorphosis of its inner structures [12]. The results will be published elsewhere.

**Figure 4:**
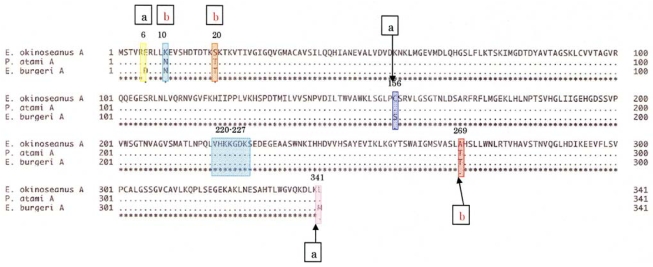
Differences in amino acid sequences of LDH-A_4_ from *E. okinoseanus*, *P. atami*, and *E. burgeri.* There were differences in six amino acid residues (6, 10, 20, 156, 269, and 341) when comparing the LDHs of the three hagfishes. a: Deep-sea type (*E. okinoseans*), b: shallow-sea type (*E. burgeri*). Reproduced with permission from the Springer Japan [11].

**Figure 5:**
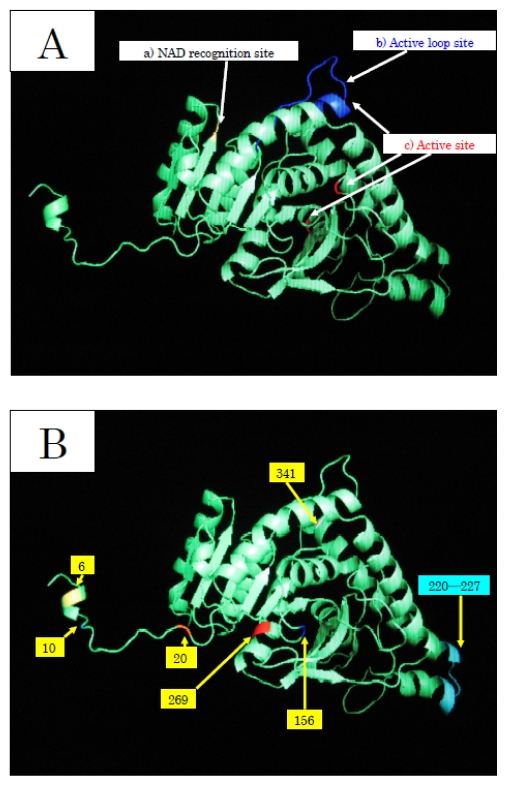
Prediction of the three-dimensional structure of hagfish LDH-A_4_. A: Functional sites of amino acid residues common to the three hagfish LDHs (a, b, c). B: Amino acid residues that vary with LDHs of the three hagfishes (6, 10, 20, 156, 269, and 341) and the region specific to LDHs of hagfish (220–227).

To elucidate the molecular mechanisms of the adaptation to high pressure, we compared the amino acid sequences (Figure 4) and three-dimensional structures (Figure 5) of LDHs in these hagfish. There were differences in six amino acid residues (6, 10, 20, 156, 269, and 341) in LDHs of the hagfishes, and additional regions specific to hagfish LDHs were found.

Two of the amino acids (156 and 269) are in the neighborhood of the active sites, and thus may control enzymatic activity. The other four amino acids (6, 10, 20, and 341) may be assigned to the part that combines four monomers into a tetramer.

The differences in the amino acid at position of the hagfish LDHs could affect their pressure resistance. Those amino acids, Asp in *E. burgeri* LDH-A_4_ and Glu in *E. okinoseanus* LDH-A_4_, have isoelectric points of 2.77 and 3.22, respectively. The deeper the habitat of the hagfish, the weaker the negative charge of the 6th amino acid. The core structures of hagfish LDHs inhabiting deeper water are only slightly affected by water. The differences in the 10th amino acid of the hagfish LDHs could affect their pressure resistance. Those amino acids are Asn in *E. burgeri* LDH-A_4_ and Lys in *E. okinoseanus* LDH-A_4_. The Lys of *E. okinoseanus* LDH-A_4_ is more hydrophilic than the Asn in *E. burgeri* LDH-A_4_, and thus the combination of four monomers into a tetramer in *E. okinoseanus* LDH-A_4_ may be tight. The N-terminal 20 residues that extend from the main body of the subunit are important in the interaction between subunits [13]. As the other amino acids (20 and 341) that are also located at the binding position in the tetramer have almost the same properties, they may not affect pressure resistance. The differences in the 269th amino acid residue of the hagfish LDHs, located in part of the core structure, could be related to the tolerance to hydrostatic pressure. The amino acids Thr in *E. burgeri* LDH-A_4_ and Ala in *E. okinoseanus* LDH-A_4_ have molecular weights of 119 and 89, respectively. The core structure of *E. okinoseanus* LDH is barely affected by hydrostatic pressure, because Ala is smaller than Thr. It can take in both pyruvate and reduced NADH, even when water enters the pocket under high hydrostatic pressure, whereas that of fish dwelling in shallow habitats cannot. The larger pocket structure would be vulnerable to high pressure. The amino acid sequences of hagfish LDHs were deduced from the nucleotide sequences obtained.

**Figure 6:**
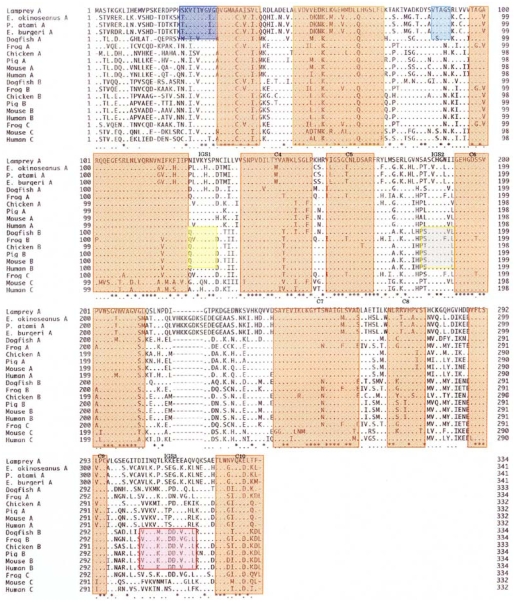
Analysis of amino acid sequences of LDH. C1–C10: Common regions, S1, S2: Cyclostomata-specific regions, IGS1-IGS3: isozyme (LDH-B) group-specific regions. Reproduced with permission from the Zoological Society of Japan [10].

The amino acid sequences of 341 residues in the skeletal muscle LDHs in the three hagfishes were compared with those of previously reported LDHs. As seen in Figure 6, the hagfish LDH-As had a specific insertion of ten amino acids (221–230). There are ten regions (C1–C10) common to LDHs from the vertebrates examined, and two Cyclostomata-specific regions (S1 and S2) have high homogeny (89–100%). Three regions (IGS1, IGS2, and IGS3) altered their structures during the differentiation of LDH isozymes, and those regions remain in vertebrate LDH-B with 67–86% homology.

**Figure 7:**
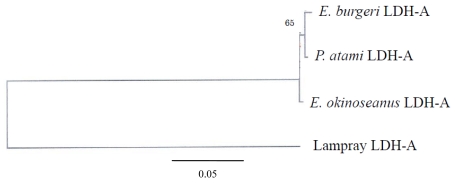
Phylogenetic tree of Cyclostomata LDHs. The tree topology and branch lengths were obtained using the neighbor-joining algorithm. The outgroup for this analysis was lamprey LDH. In this case, 1000 bootstrap pseudoreplications were analyzed, and the number near the node indicates the percentage of optimal trees in which this node appeared; values <50% are not shown. The scale at the bottom of this figure indicates branch length in substitutions per site. Reproduced with permission from the Zoological Society of Japan [10].

The phylogenetic tree of Cyclostomata LDH genes inferred from the present results suggests that LDH-A of hagfish diverged just after that of lampreys (Figure 7). Although they probably evolved from a single ancestral gene, the genes encoding LDH isoenzymes are now quite distinct. The mechanism of evolution of LDH-A to LDH-B may be clarified through further investigation of the most prototypical LDH-B in hagfish.

**Figure 8:**
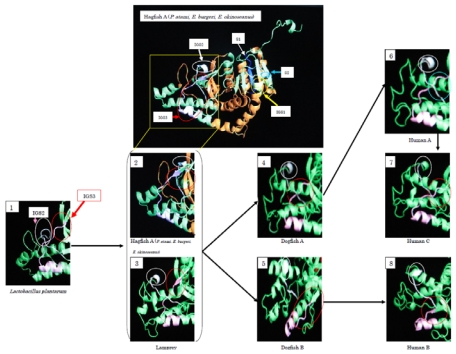
Three-dimensional structures of LDH. Reproduced with permission from the Zoological Society of Japan [10].

During the evolution from Bacteria to Cyclostomata, residues 1–22 at the *N*-terminus were added. IGS2 varies from a loop structure to a β-sheet structure. At the same time, IGS3 changed from a loop structure into a β-sheet, and it changed again to a loop structure during the evolution to dogfish LDH-A. The β-sheet structure remains in LDH-B. Fifty-seven percent of the amino acids of cyclostomata IGS3, 43% of dogfish LDH-A, 86% of dogfish LDH-B, and 14% of human LDH-A are in a β-sheet, while all (100%) of human LDH-B amino acids are postulated to be in a β-sheet. The three-dimensional structures presented in Figure 8 suggest that Cyclostomata IGS3 has properties intermediate between those of A and B before it differentiates into the A and B types. This tendency was similar in IGS1 and IGS2.

## 3. Experimental Section

### 3.1. Materials

*E. okinoseanus* and *P. atami* were collected from Suruga Bay off Shizuoka, Japan. The sampling locations were at 34–35′ N, 138–139′ E, at a depth of approximately 700–1000 m for *E. okinoseanus* (ambient seawater temperature 5–8 °C) and at a depth of approximately 250–400 m for *P. atami* (ambient seawater temperature 10 °C). *E. burgeri was* collected in an inlet off Misaki in Kanagawa, Japan. The sampling location was 35.8′ N, 139.37′ E, at a depth of approximately 45–60 m (ambient seawater temperature 10 °C). *E. japonicus was* collected in the Shiribetu River in Hokkaido, Japan. The sampling location was 42.5′ N, 140.35′ E. Skeletal muscles and hearts of the Cyclostomata were washed with PBS buffer (137 mM NaCl, 8.10 mM Na_2_HPO_4_, 2.68 mM KCl, 1.47 mM KH_2_PO_4_, pH 7.4) pretreated with diethylpyrocarbonate to remove RNase and were frozen immediately at −80 °C.

### 3.2. Methods

mRNAs were isolated from homogenates of skeletal muscle following the method of Loening [14]. Total cDNA was subjected to PCR with the following eight primers:

- F1, GTGACAATAGTTGGAATCGG; F2, GTGGTTTGTGCTTCACACGA;
- F3, TGAACCTTGTACAGAGGAAC; R1, CCACTCCATACAGGCAC;
- R2, AGCAGGATTTCCTCCCACTAC; R3, GTTCCTCTGTACAAGGTTCA;
- R4, CTTCCTGTTGTCTAACTCCA; and R5, CCGATTCCAACTATTGTCAC.

The primers were based on the nucleotide sequences of active sites of human, pig, mouse, and frog LDHs. PCR amplification of the coding sequence with primer pairs F1-R1 and F2-R2 was performed. A typical thermal cycling profile consisted of 40 cycles of the following three steps: denaturation at 94 °C for 0.5 min; annealing at 50 °C for 0.5 min; and extension at 72 °C for 1 min. The cycling steps were preceded by a 2-min denaturation at 94 °C and followed by a 7-min extension at 72 °C. The remaining portions of the coding sequence and the 5′ and 3′ untranslated regions were amplified using the 5′- and 3′-rapid amplification of cDNA ends (RACE) procedures. In 5′-RACE, primer R3 was used for reverse transcription, and primers R4 and R5 served as the gene-specific primers for PCR amplification. Primer F3 was used for 3′-RACE. The PCR products were purified with 2% agarose gel electrophoresis and subcloned into the pTZ19R vector. After the transformation of these recombinants into XL1-Blue using the calcium chloride method, plasmid DNA was recovered and their nucleotide sequences were determined using a DNA Sequencer (Model 4000, LI-COR Inc., Lincoln, NE, USA).

Isozymes of LDH were detected as follows. After electrophoresis of the homogenized tissues (skeletal muscle and heart) on 1% agarose gels for 0.5 h at 250 volts, LDH activity bands were visualized using the following staining solution [15]: 28 mg NAD, 1.3 mg diaphorase, and 3.5 mg *p*-nitroblue tetrazolium chloride in 3 ml of 0.05 mol/l sodium lactate–0.14 mol/l Tris-citrate buffer (pH 8.6).

The amino acid sequences of LDHs were deduced from the nucleotide sequences obtained. Homology search analysis was performed using the GENETYX software system (Software Development Co., Ltd., Tokyo, Japan). The phylogenetic tree was inferred using the neighbor-joining method [16]. Three-dimensional models were produced with the 3D-JIGSAW modeling program (Cancer Research UK, London, England) [17].

The skeletal muscles of the three hagfish species were homogenized in a 7-fold dilution of 0.25 M sucrose solution, and LDH was purified with DEAE-cellulose and 5′-AMP-Sepharose 4B after desalting with (NH_4_)_2_SO_4_ and dialysis as described by Brodelius *et al.* [18] with minor modifications. The sample obtained was confirmed to be a single peptide band upon disc gel electrophoresis [19] using 7% acrylamide gel stained with Coomassie Brilliant Blue [20]. The disc was dipped in the reaction mixture described below, and the LDH activity in the band was visualized based on the absorption change of formazan added to the solution following the method of Kuroda and Yoshida [15]. Protein concentrations were determined using the Bio-Rad (Hercules, CA, USA) protein assay kit I according to the manufacturer’s instructions. Bovine serum albumin was used as a standard.

The conversion of pyruvate to lactate by LDH was determined as described by Amador *et al.* [21] with a slight modification in the optimum pH and temperature for hagfish, *i.e.*, pH 6.2 and 30 °C. Ten microliters of LDH solution (6.0 μg/ml) was added to the reaction mixture (0.83 mM pyruvate, 0.13 mM NADH, 0.1 M MES buffer, pH 6.2), and changes in the absorbance at 340 nm (A_340_) were monitored using a spectrophotometer (Shimadzu 1600PC, Kyoto, Japan) equipped with a high-pressure optical cell [22]. The activity was determined based on the absorption decrease at 340 nm within 3 min after the addition of LDH under various pressure conditions. The Hill unit is the amount of LDH which changes the optical density of NADH at 340 nm by 0.001 in 30 min [23]. The cDNA of LDHs was determined, and the DDBJ accession numbers of the hagfish LDHs from *E. okinoseanus*, *P. atami*, and *E. burgeri* are AB369246, AB369247, and AB369248, respectively.

## 4. Conclusions

The LDHs of the skeletal muscle from the three hagfishes *E. burgeri*, *E. okinoseanus*, and *M. garmani*, which are the lowest extant vertebrates, were purified and their properties were examined. The hagfish were revealed to have 10 specific additional amino acids, for a total of 341. The additional sequence (221–230) is located next to the hinge position (219) to introduce NAD and pyruvate. The additional sequence may cause less flexibility at the hinge position, resulting in the heat instability of hagfish LDHs. The optimal pH values (8.5–9.5) in the reaction from lactic acid to pyruvic acid of hagfish LDHs were almost the same as those of human (8.8) and cod (9.0) LDHs, although the optimum pH values (6.0–6.2) in the reaction from pyruvic acid to lactate acid of hagfishes were more acidic than those of human (7.2) and cod (7.5) LDHs. Differences in the three hagfish LDHs were apparent in the direction of the reversible reactions.

In the Cyclostomata, *E. japonica* has been shown to have a single LDH subunit, while hagfish have two subunits, LDH-A and LDH-B. Thus, hagfish LDH-B is the most primitive form examined. During the evolution from bacteria to lampreys, residues 1–22 at the *N*-terminus were added. This region is the part where the subunits connect with each other and changes from hydrophobic to hydrophilic during evolution. It is assumed that changes in this part caused the diversification of the isozymes.

The effects of high hydrostatic pressure on LDH activity were examined under pressures from 0.1 to 100 MPa. The activity of LDH-A_4_ from *E. burgeri* (shallow-sea species) was lost at pressure greater than 5 MPa. The activity of LDH-A_4_ from *P. atami* (inhabiting depths of ~400 m) was maintained at pressure of 10 MPa, but decreased to about 55% at 15 MPa and to about 15% at 20 MPa. Among the three hagfish, *E. okinoseanus* inhabits the deepest waters at around 800–1000 m. Its LDH-A_4_ appeared to be fully active at pressures up to 40 MPa and it maintained 70% of that activity even at 100 MPa. These results suggest that the LDH-A_4_ of *E. okinoseanus* is more adapted to high-pressure conditions than that of the other hagfishes.

Three possible mechanisms can be proposed as the reason for the inactivation of hagfish LDHs at high hydrostatic pressure: (1) the dissociation of the LDHs into four monomers; (2) changes in the structures near active sites; and (3) an increase in the activation volumes of LDHs. Six amino acid substitutions between the three LDH primary structures were found. Among them, two amino acid residues are likely to be responsible for the high-pressure resistance of *E. okinoseanus* LDH. On the LDHs from both deep-sea and shallow living species, *Corphaenoides armatus* and *Gadus morhua*, the results of circular dichroism spectroscopic analysis were reported protein unfolding as the cause of inactivation at high pressure [9]. The differences in the 6th amino acid residue of the hagfish LDHs would affect their pressure resistance. The results of electrophoresis under high-pressure conditions show that the dissociation of tetramers caused the inactivation of *E. burgeri* LDH [12]. To confirm that the predicted effects of substitutions indeed occur, we are performing site-directed mutagenesis. This method is the replacement of Ala6 with Glu in *E. okinoseanus* LDH (now in progress).

Although the cDNAs of the LDH-A of the three hagfishes were clarified in the genetic study, hagfish LDH-B, which diverged from the lamprey LDH and achieved molecular variety for the first time, has not been elucidated. Thus, the amino acid sequences of those LDH-B and their enzymatic properties should be examined. The phylogenetic tree of Cyclostomata LDH-A genes was demonstrated in this study, but the relationship between Cyclostomata LDH and those of the Bacteria is not yet completely understood. The LDH-As of both the Ascidians and the Amphioxus, which are located just before the Cyclostomata in the phylogenetic tree, would be key in the study of evolution. The compositions of LDH isozymes are reported to change in human diseases [24]. The relationship between the pressure tolerance of LDH and its isozyme composition is expected to contribute to the diagnosis of some human diseases.

**Figure 9:**
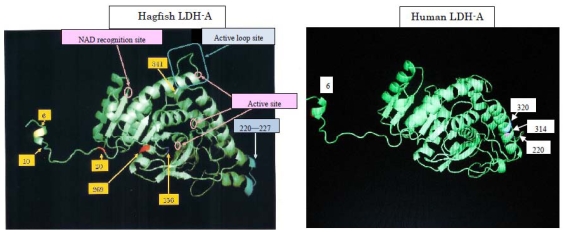
Three-dimensional structures of hagfish LDH-A and human LDH-A. Reproduced with permission from the Ogata Institute for Medical and Chemical Research [25].

Evolutionary medicine studies diseases from the viewpoint of evolution. We propose herein a new field, molecular evolutionary medicine, which focuses on genetic evolution [25]. We are comparing genetic variations in relation to human diseases. As an example, human and hagfish LDHs are related (Figure 9). When subjected to physical pressure, those LDHs exhibit new properties. The amino acids responsible for pressure tolerance are the same in both human and hagfish LDHs, and substitutions of these amino acids occurred as adaptations during evolution. Mutations in these amino acids can result in anomalies that may be implicated in the development of human diseases. We are developing a device that can measure LDH activity in the blood of the patient with 6th amino acid mutation at around three MPa, which is below the pressure causing the dissociation of rabbit LDH-As and pig LDH-Bs (data not shown).
